# 

**Published:** 1883-09

**Authors:** 


### CONTENTS:

Art. I.—Transactions of the State Boards of Dental Examiners.... 193
Art. II.—General Anæsthesia Scarcely Justifiable in Extraction of Teeth. By J. Hardeman, D. D. S.... 207
Art. III.—Tartar and its Removal from the Teeth. By Theo. F. Chupein, D. D. S.... 213
Art. IV.—On Certain Conditions of Dead Teeth. By Chas. E. Tomes.... 219
Art. V.—Bromide of Ethyl the most Perfect Anæsthetic for Short Operations. By Prof. J. J. Chisolm, M D.... 224
Art. VI.—Simplified Treatment of Diseases of the Dental Pulp, by Iodoform, Etc. 228
Art. VII.—On the Conservative Treatment of the Dental Pulp. By T. Charters White, D. D. S.... 230
Art. VIII.—The Teeth from a Medico-Legal Aspect.... 233
Maryland and District Columbia Association Meeting.... 235

### EDITORIAL, ETC.

The Dental Association Meetings.... 236
The University of Maryland.... 237

### MONTHLY SUMMARY.

Successful Exsection of Inferior Dental Nerve.... 238
Hot Water as a Restorative in Chloroform Narcosis.... 239
Syphilis and Rachitis.... 240
Inodorous Iodoform.... 240
New Discoveries.... 240

